# The Dynamic Accumulation Rules of Chemical Components in Different Medicinal Parts of *Angelica sinensis* by GC-MS

**DOI:** 10.3390/molecules27144617

**Published:** 2022-07-20

**Authors:** Yuying Chen, Qian Li, Daiyu Qiu

**Affiliations:** State Key Laboratory of Aridland Crop Science, College of Agronomy, Gansu Agricultural University, Lanzhou 730070, China; chenyuying5676@163.com (Y.C.); qiudy@gsau.edu.cn (D.Q.)

**Keywords:** *Angelica sinensis*, different medicinal parts, accumulation rules

## Abstract

The chemical components and medicinal properties of different medicinal parts of *Angelica sinensis* are often used as medicine after being divided into the head, body and tail of *Angelica sinensis*. In this study, the chemical components of different medicinal parts in different periods were analyzed by GC-MS for the first time, and the differences of the accumulation rules of chemical components in different medicinal parts of *Angelica sinensis* were obtained. This study demonstrated that the differences of composition accumulation in different medicinal parts of *Angelica sinensis* were mainly reflected in the types and relative contents of compounds. The study found that the number of compounds in different medicinal parts of *Angelica sinensis* in each period were different and the change rules of the same compound in different medicinal parts were also different. The number of compounds in the tail of *Angelica sinensis* was the least in April, and the largest in October. The content of ligustilide in the body of *Angelica sinensis* was higher in April and was the highest in the tail in October. The relative content of butylidenephthalide in the head was the highest in October. The relative contents of senkyunolide A and butylphthalide in the head were decreased in October, while the contents in the body and tail increased, indicating that the compounds that accumulate in the head may transfer to the body and tail in later stages of growth. This study clarified the differences in the accumulation of chemical components in different medicinal parts of *Angelica sinensis*, which could provide a theoretical basis for the reasons for the differences of chemical components in the different medicinal parts.

## 1. Introduction

*Angelica sinensis* Radix (*A. sinensis*) is the dried root of the *Angelica Sinensis* (Oliv.) Diels [1]. It has the effect of replenishing blood, activating blood circulation and regulating irregular menstruation [2,3]. During the Ming and Qing Dynasties, doctors clearly divided *A. sinensis* into head (the upper part of the root), body (the taproot) and tail (lateral roots). It is divided into different parts and used as medicine. The main function of the head is to stop bleeding, the body has a strong effect of replenishing blood and the tail focuses on promoting blood circulation and removing blood stasis. There is a high correlation between the difference in the medicinal properties of the different parts of *A. sinensis* and the basis of the chemical substances [4]. Studies have found that the content of the same substance in the different medicinal parts was different, the content of volatile oil and ferulic acid in the tail of *A. sinensis* was the highest, and the content of total tannins in head was the highest [5]. Other researchers determined the content of ferulic acid in different medicinal parts of *A. sinensis* by HPLC and also reached the conclusion that the content of ferulic acid in the tail of *A. sinensis* was the highest [6].

The accumulation dynamics of chemical components in medicinal plants is closely related to growth and development. *A. sinensis* is a perennial herb with a total growth cycle of three years with two winters (about 800 days), mainly in three stages: grow–seedling stage, medicine formation period, and reserve–seed period [7]. The first year is the grow–seedling stage, which mainly increases the length of roots to meet the needs of water and inorganic salts in the later growth process [8]. At this stage, the content of Z-ligustilide gradually increased and Z-butenylphthalide decreased; in the early stage of the second year, the stems and leaves grew vigorously, and the accumulated assimilates were transported to the roots through photosynthesis. The contents of Z-ligustilide and Z-butenylphthalein decreased with the growth of stems and leaves and gradually increased after the stems and leaves began to wither. During the reserve–seed period in the third year, the biological yield gradually decreased: these two components first increased with the growth of stems and leaves and then decreased sharply to the lowest at the bolting and flowering stages. In the process of seed maturation to harvest, the content increased briefly and then decreased [9,10,11,12,13,14].

There are obvious differences in the chemical components of different medicinal parts of *A. sinensis*, and the components and pharmacological activities change greatly in different periods [15]. At present, there is no report on the accumulation law of chemical components in different medicinal parts of *A. sinensis* in different periods. Therefore, GC-MS technology was used to study the accumulation dynamics of different medicinal parts of *A. sinensis* and to analyze the differences of chemical components in different medicinal parts in each period to clarify the rules of chemical component accumulation and mutual transformation in the head, body and tail of *A. sinensis*.

## 2. Materials and Methods

### 2.1. Materials and Reagents

The plant materials of *A. sinensis* used in this experiment were fresh materials that were collected from Xizhai village, Xizhai Town, Min County, Dingxi City, Gansu Province on April 15 (seedling stage), June 6 (leaf growing stage), August 24 (root enlargement stage), September 10 (late root expansion stage) and October 24 (harvest stage) in 2021 and were brought back in an ice box. The voucher specimens (No. GAUAB-AS-20210415, No. GAUAB-AS-20210606, No. GAUAB-AS-20210824, No. GAUAB-AS-20210910 and No. GAUAB-AS-20211025) were deposited in the herbarium of Department of Chinese herbal medicine, Agronomy building of Gansu Agricultural University, Lanzhou, China. The n-Hexane (Lot 110-54-3, Chromatographic grade) was purchased from Shenzhen Dongmao Chemical Reagent Co., Ltd. (Shenzhen, China).

### 2.2. Sample Solution Preparation

**Figure 1:**
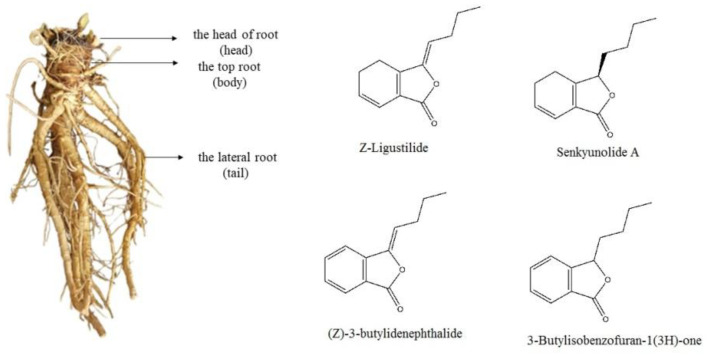
The photo of *A. sinensis* root and structures of main components.

The fresh materials in different periods were divided into head, body and tail (see the left photo of Figure 1). They were subsequently dried to constant weight in a freeze dryer and then crushed. The n-hexane (25 mL) was added to the sample prepared by precisely weighing 2.5 g of the plant material and soaking for 1 h, then ultrasonically extracted for 30 min, the extraction repeated twice and then the filtrate was combined. The combined filtrate was concentrated at 40 °C for 5 min in a rotary evaporator, the concentrate dissolved with n-hexane and adjusted to 5 mL. Finally, it was filtered with a 0.22 μm microporous membrane to obtain *A. sinensis* sample solution [16].

### 2.3. GC–MS Conditions

#### 2.3.1. GC–MS Column

The column used was a DB-23 (30 m × 0.25 mm × 0.25 μm). The carrier gas was high purity helium, and its flow rate was 1.0 mL/min. The initial temperature was maintained at 60 °C for 3 min, and then raised to 270 °C at a rate of 10 °C/min. All samples were injected in split mode at 270 °C. The split injection was 5:1.

#### 2.3.2. Mass Spectrum Conditions

An electron impact ion source was used, with full scanning mode (mass range m/z 50–650), ion source temperature 230 °C, interface temperature 250 °C, quadrupole temperature 150 °C, electronic energy 70 eV and solvent delay time 3 min. The ion detection mode selected ion monitor was selected.

### 2.4. Data Analysis

GC-MS was used to perform a full ion scan of the compound, and the total ion current map of different medicinal parts of *A. sinensis* in different periods was obtained. The compound was qualitatively analyzed by searching the NIST14 standard mass spectrometry library, and according to the peak area normalization method the relative content of each component was calculated.

## 3. Results and Discussion

### 3.1. Analysis of Chemical Components in the Head of A. sinensis in Different Periods

**Figure 2:**
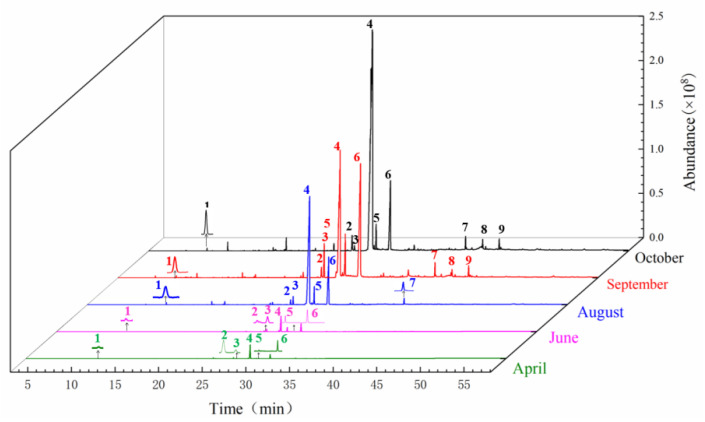
Total ion chromatogram from the head of root of *A. sinensis* in different periods. Note: 1: α-Pinene; 2: Z-Butylidenephthalide; 3: 3-Butylisobenzofuran-1(3H)-one; 4: (E)-Ligustilide; 5: Senkyunolide A; 6: (Z)-Ligustilide; 7: Panaxynone; 8: γ-Sitosterol; 9: Senkyunolide H.

The sample solution of the head of *A. sinensis* in different periods was analyzed by GC-MS. The GC-MS chromatogram in different periods was shown in Figure 2. The chemical composition and relative concentrations were obtained using the peak area normalization method (Table 1).

A total of 12 compounds were identified in April: (Z)-ligustilide, (E)-ligustilide, 3-butylisobenzofuran-1(3H)-one, senkyunolide A, α-pinene, etc., among them, the component with the highest relative content was (Z)-ligustilide, which was 1.16%. In June, 20 components were identified, 3-carene, p-cymene, azulene, α-himachalene, panaxynone, ethyl iso-allocholate, 6-undecanone and tetradecane were newly added compounds. A total of 42 compounds were identified in August, mainly increasing α-acorenol, β-copaene, β-chamigrene, patchoulene, β-bisabolene, (+)-bicyclogermacrene, α-longipinene, trans-isoeugenol and other phenolic compounds. In September, 11 compounds including (9Z,12Z)-9,12-Octadecadienoic acid, (Z)-9-octadecenoic acid methyl ester and β-farnesene were newly added. In October, phenolic compounds such as cis-β-ocimene, β-ylangene, thujopsene-(I2), phthalides and vitamin E were added. It was found that August was the peak period of accumulation of phenolic compounds, oleic acid compounds accumulated more in September, and October was the period of accumulation of rare volatile oil compounds; it was also the key period of rapid increase in content.

**Figure 3:**
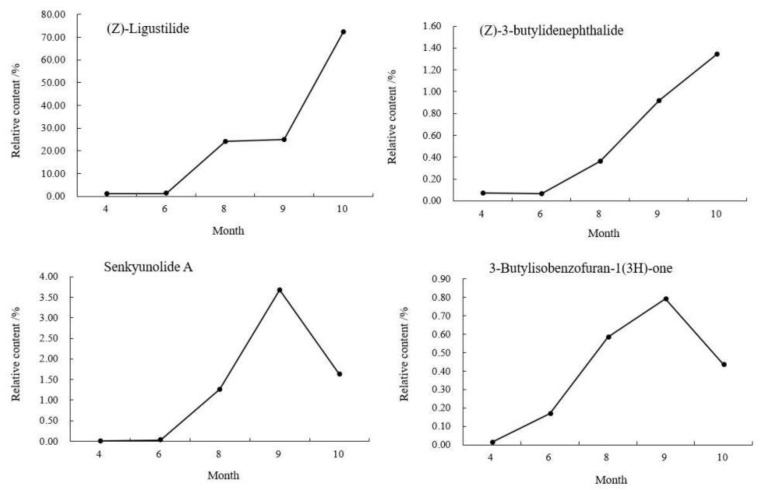
Changes of chemical components in the head of *A. sinensis* in different periods.

The relative contents of the compounds in different periods were analyzed by the peak area normalization method, and the accumulation dynamics of four important active components (see the right photo of Figure 1) in the head of *A. sinensis* were analyzed. As shown in Figure 3, the relative content of (Z)-ligustilide increased gradually from April to October and increased rapidly from September to October. The relative content of (Z)-3-butylidenephthalide decreased by 0.0082% in June, and then showed a gradual upward trend. The relative contents of senkyunolide A and 3-butylisobenzofuran-1(3H)-one reached the maximum value of 3.6762% and 0.7921% in September, and the contents decreased in the harvest period. Combining the changes of compounds in each period, (Z)-ligustilide was the highest content component from seedling stage to harvest stage, and the relative content was up to 72.2466% in harvest stage, which was the main core component in the head of *A. sinensis* in each period.

### 3.2. Analysis of Chemical Components in the Body of A. sinensis at Different Periods

**Figure 4:**
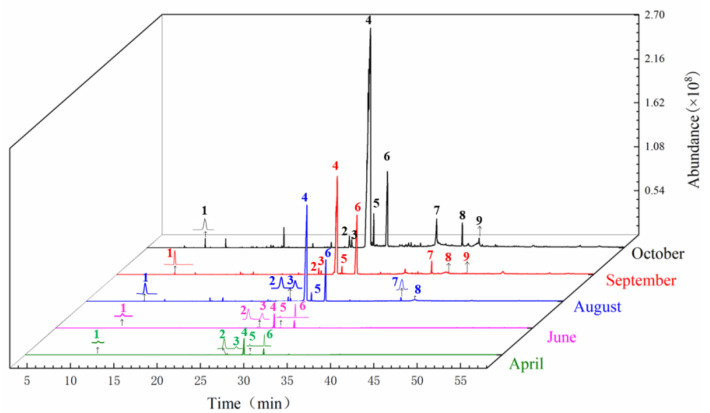
Total ion chromatogram from the body of *A. sinensis* in different periods. Note: The compounds represented by peaks 1~9 are the same as the compounds in Figure 2.

The GC-MS chromatogram of *A. sinensis* of the body of *A. sinensis* in different periods was shown in Figure 4. The chemical composition and relative concentrations were obtained using the peak area normalization method (Table 2). The types of chemical components in the body of *A. sinensis* varied greatly in different periods.

In April, the compounds in the body were the same as those in the head of *A. sinensis*. They mainly contain Z-ligustilide, (Z)-3-butylidenephthalide, senkyunolide A and other ester compounds and a small amount of olefin compounds. In June, 12 components were added, and 4 compounds were added compared with the head in the same period: 1,2,6,6-tetramethylcyclohexa-1,3-diene, α-acorenol and trans-Isoeugenol. A total of 48 components were identified in the body of *A. sinensis* in August. In addition to a large number of phenolic components added in the same period, vitamin E, γ-sitosterol, (Z,Z)-9,12-octadecadienoic acid, (3R,3aR,7R,8aS)-3,8,8-trimethyl-6-methyleneoctahydro-1H-3a,7-methanoazulene, etc. were also added. In September, 11 new compounds were added in the body of *A. sinensis*, mainly including cis-β-ocimene, thujopsene-(I2), (+)-cuparene, 11,14-eicosadienoic acid-methyl ester, carveol and 9-hexadecenoic acid. In October, 68 compounds were identified, and the relative content of Z-ligustilide was the highest, at 71.3681%. Seven new compounds were added, including β-ylangene, 9-hexadecenoic acid, cis-5,8,11,14,17-eicosapentaenoic acid and campesterol.

**Figure 5:**
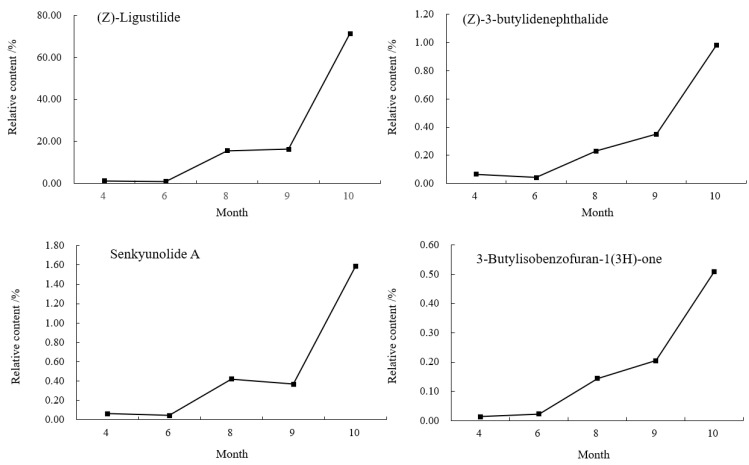
Changes of chemical components in the body of *A. sinensis* in different periods.

As shown in Figure 5, it was found that from April to June, the contents of Z-ligustilide, (Z)-3-butylidenephthalide and senkyunolide A in the body of *A. sinensis* decreased by 0.2584%, 0.0235% and 0.0183%, 3-butylisobenzofuran-1(3H)-one increased by 0.0092%. The aboveground part grew vigorously in June, and these three reduced compounds might be involved in the transformation of the compounds when the aboveground part grew. The content of senkyunolide A decreased by 0.0525% in September; the other three compounds showed an increasing trend from June to October, entered a rapid accumulation period in September and reached the maximum value in October. The relative contents of Z-ligustilide, (Z)-3-butylidenephthalide, senkyunolide A and 3-butylisobenzofuran-1(3H)-one were: 71.3681%, 0.9806%, 1.5863% and 0.5095%.

### 3.3. Analysis of Chemical Components in the Tail of A. sinensis at Different Periods

**Figure 6:**
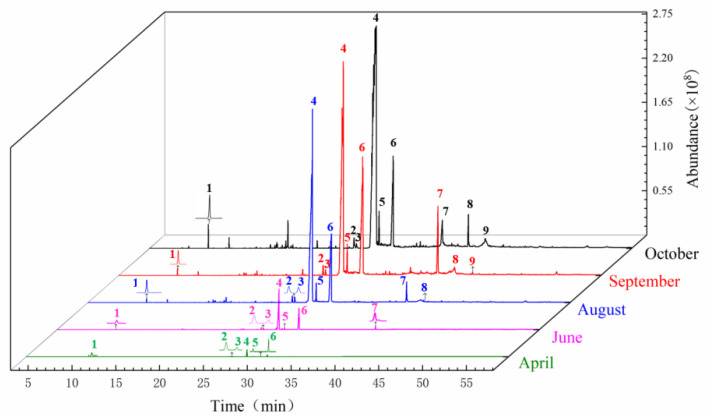
Total ion chromatogram from the tail of *A. sinensis* in different periods. Note: The compounds represented by peaks 1~9 are the same as the compounds in Figure 2.

The GC-MS chromatogram of *A. sinensis* of the tail of *A. sinensis* in different periods was shown in Figure 6. The chemical composition and relative concentrations were obtained using the peak area normalization method (Table 3).

In April, a total of 11 compounds such as (Z)-ligustilide, (E)-ligustilide, (Z)-3-butylidenephthalide, senkyunolide A, α-pinene, arteannuin b, etc. were identified in the tail of *A. sinensis.* Compared with April, 20 new compounds were added in June, such as 3-carene, β-copaene, β-chamigrene, α-himachalene, p-cymene, (Z)-9-octadecenoic acid, methyl ester, 6-undecanone, panaxynone, carveol, α-acorenol, etc. A total of 55 compounds were identified in August, including olefin compounds such as cis-β-ocimene, cis-β-farnesene, β-bisabolene, α-longipinene, limonen-6-ol, pivalate, 2,5-Octadecadiynoic acid, methyl ester, (Z,Z)-9,12-Octadecadienoic acid ethyl ester and organic acids such as 9-Hexadecenoic acid, (Z,Z)-9,12-Octadecadienoic acid, etc. In September, 7 new compounds, such as thujopsene-(I2) and (+)-cuparene were added. A total of 69 compounds were identified in October, and 7 new compounds were added: including 9-hexadecenoic acid, 9-octadecen-12-ynoic acid methyl ester, 10,13-octadecadiynoic acid methyl ester, pentylbenzene, α-elemene, β-guaiene, and β-ylangene.

**Figure 7:**
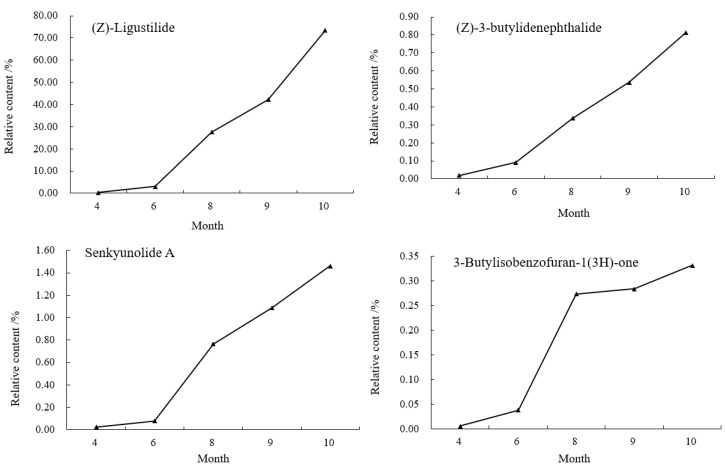
Changes of chemical components in the tail of *A. sinensis* in different periods.

As shown in Figure 7, in the tail of *A. sinensis*, Z-ligustilide, (Z)-3-butylidenephthalide, senkyunolide A and 3-butylisobenzofuran-1(3H)-one were also selected to analyze the relative content change trend. The results showed that the contents of these four compounds showed a gradual upward trend from April to October. The growth was relatively slow from April to June, entered the period of rapid growth after June, and reached the highest value in October. The relative contents were 73.4925%, 0.8135%, 1.4591% and 0.3314%, respectively.

### 3.4. Analysis of the Differences in the Accumulation of Compounds in Different Medicinal Parts of A. sinensis

The number of compounds identified in different medicinal parts of *A. sinensis* in different periods was shown in Table 4. From April to October, the number of compounds in the head, body and tail of *A. sinensis* showed an increasing trend. In April, there was one less compound in the tail than in the head and body. In June, there were 11 and 7 more compounds in tail than head and body, respectively. It mainly contained olefin compounds such as β-chamigrene and 1,2,6,6-tetramethylcyclohexa-1,3-diene and ester compounds such as (Z)-9-octadecenoic acid, methyl ester and 7,10-octadecadienoic acid, etc. Since then, the species of compounds in the tail of *A. sinensis* were always the highest. During the harvest period, the number of species gradually reached a balance, and the number of compounds in head, body and tail were 61, 68 and 69, respectively, and there were differences in the types of components. In the harvest period, 6-epi-shyobunol only existed in the head of *A. sinensis*, eicosapentaenoic acid was found only in the body of *A. sinensis*, and 10,13-Octadecadiynoic acid and α-Elemen were only found in the tail of *A. sinensis*. 9-Hexadecenoic acid, 9-Octadecen-12-ynoic acid methyl ester, limonen-6-ol, pivalate, (+)-cuparene, carveol and (Z,Z)-9,12-octadecadienoic acid were not found in the head but existed in body and tail of *A. sinensis*.

**Figure 8:**
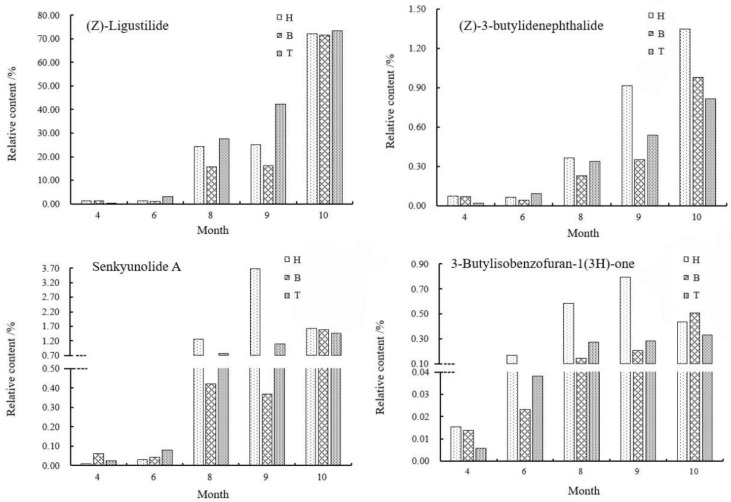
Changes of chemical components in different medicinal parts in different periods. Note: H, head; B, body; T, tail.

The relative content changes of the four main active components in *A. sinensis* in different medicinal parts were shown in Figure 8. In April, the content of Z-ligustilide in the body of *A. sinensis* was the highest, 0.8323% higher than that in the tail. From June, the content of Z-ligustilide in the tail was gradually higher than that in the head and body. From June to October, the relative content remained as tail > head > body. During the rapid accumulation of content in September, the difference of relative content in head, body and tail gradually decreased and tended to balance.

As one of the important active components in *A. sinensis*, the accumulation law of (Z)-3-butylidenephthalide was different from that of Z-ligustilide. In April, the relative content of (Z)-3-butylidenephthalide in the head was higher, which was 0.0159% higher than that in the tail. In August, the content in the head was higher, and from August to October, the relative content gap between the head and the body and the tail gradually increased, and the highest content in the head was 1.3462% during the harvest period, which was 0.5327% higher than that in the tail. Researchers [17] analyzed the chemical components of different parts by GC-MS and found that the relative contents of compounds in different parts were different. Z-ligustilide was the main compound, which was consistent with the results of our study. Ligustilide compounds affect platelet aggregation or thrombosis [18], and the content of Z-ligustilide in tail was the highest, which was consistent with the effect of tail focusing on promoting blood circulation.

In April, the content of senkyunolide A in the body was higher; the content in the head had always been the highest since August and reached the highest value of 3.6762% in September. In October, the content in the head of *A. sinensis* decreased by 2.0498%, while the content in the body and tail increased by 1.2184% and 0.3708%, respectively, which may be caused by the transfer of senkyunolide A from head to body and tail.

From April to September, the content of 3-Butylisobenzofuran-1(3H)-one in the head was always the highest and decreased by 0.3047% in the head and increased by 0.3046% and 0.0477% in the tail by the harvest period. The relationship of the content of 3-Butylisobenzofuran-1(3H)-one at the harvest period was: body > head > tail. In September, the aboveground parts withered gradually, the required nutrients decreased gradually and a large number of assimilates transferred to the roots. At this time, the contents of (Z)-3-butylidenephthalide, senkyunolide A and 3-butylisobenzofuran-1(3H)-one decreased in the head and increased in the body and tail, which can confirm this inference. It was speculated that there was a phenomenon that the compounds that accumulated in the head transferred to the body and tail during the accumulation of effective components in different medicinal parts. Overall, the accumulation and distribution of compounds were consistent with the efficacy of different medicinal parts of *A. sinensis* and the accumulation of the types and contents of the compounds reached the maximum in October, which was consistent with the traditional harvesting period [19].

The content changes of the same compound in different medicinal parts of *A. sinensis* in different periods may be caused by some related factors during growth and development such as environmental factors, temperature and solar radiation, which would accelerate the synthesis of a compound, thus slowing down the synthesis of other related compounds [20,21]. But the specific mechanism is not clear at present.

## 4. Conclusions

In summary, we revealed the differences of accumulation dynamics in different medicinal parts of *A. sinensis* by GC-MS for the first time, which were mainly reflected in the component types and relative contents. The number of compounds contained in different medicinal parts of *A. sinensis* showed an increasing trend from April to October, but the number of compounds in different medicinal parts was different in each period and the types of components were also different. This study revealed the accumulation rule of chemical constituents in different medicinal parts of *A. sinensis* and provided a theoretical basis for the differences in compounds and medicinal properties in different medicinal parts.

## Figures and Tables

**Table 1:** Analysis of chemical components in the head of *A. sinensis* in different periods.

Number	Compounds	Formula	Relative Content (%)				
			April	June	August	September	October
1	3-Carene	C_10_H_16_	-	0.0024	0.0062	0.0738	0.034
2	α-Pinene	C_10_H_16_	0.0015	0.0031	0.0202	0.0686	0.0988
3	cis-β-Ocimene	C_10_H_16_	-	-	-	-	0.0037
4	1,2,6,6-tetramethylcyclohexa-1,3-diene	C_10_H_16_	-	-	-	-	0.0065
5	5-Pentylcyclohexa-1,3-diene	C_11_H_18_	0.003	0.0013	0.0836	0.1578	0.3001
6	Pentylbenzene	C_11_H_16_	-	-	-	-	0.0074
7	p-Cymene	C_10_H_14_	-	0.0008	0.003	0.0173	0.0046
8	Benzeneacetaldehyde, α-ethyl-	C_11_H_16_	-	-	0.0033	0.0082	0.0072
9	Azulene	C_10_H_8_	-	0.0003	0.0032	0.0092	0.0027
10	3-methyl-5-propylnonane	C_13_H_28_	-	-	0.0059	0.0213	0.0249
11	Naphthalene, 1,2,3,4-tetrahydro-1-methyl-	C_11_H_14_	-	-	-	0.0032	0.0026
12	6-Undecanone	C_11_H_22_O	-	0.0013	0.0088	0.0193	0.0323
13	Elemene isomer	C_15_H_24_	-	-	-	-	0.0284
14	2,5-Octadecadiynoic acid, methyl ester	C_19_H_30_O_2_	-	-	0.0017	0.0067	0.0052
15	Tetradecane	C_14_H_30_	-	0.0023	0.0092	0.0428	0.0519
16	Bicyclo[4.4.1]undeca-1,3,5,7,9-pentaene	C_11_H_10_	-	-	0.0028	0.0065	-
17	Dicyclopentadiene diepoxide	C_10_H_12_O_2_	-	-	-	0.0069	0.0086
18	α-acorenol	C_15_H_26_O	-	-	0.0023	0.0075	0.0067
19	(3R,3aR,7R,8aS)-3,8,8-Trimethyl-6-methyleneoctahydro-1H-3a,7-methanoazulene	C_15_H_24_	-	-	-	0.0334	0.0654
20	β-copaene	C_15_H_24_	-	-	0.0032	0.0049	0.011
21	Thujopsene-(I2)	C_15_H_24_	-	-	-	-	0.0036
22	2-Methoxy-4-vinylphenol	C_9_H_10_O_2_	0.0101	0.0061	0.1542	0.223	0.1538
23	β-Farnesene	C_15_H_24_	-	-	-	0.0404	0.0668
24	(±)-Gymnomitrene	C_15_H_24_	-	-	-	-	0.0706
25	α-Himachalene	C_15_H_24_	-	0.002	0.0349	0.082	0.0703
26	6-epi-shyobunol	C_15_H_26_O	-	-	-	-	0.0269
27	β-Chamigrene	C_15_H_24_	-	-	0.0083	0.016	0.0331
28	Patchoulene	C_15_H_24_	-	-	0.0024	0.0037	0.0123
29	β-Guaiene	C_15_H_24_	-	-	-	-	0.0096
30	β-Bisabolene	C_15_H_24_	-	-	0.0119	0.0246	0.0531
31	(+)-Bicyclogermacrene	C_15_H_24_	-	-	0.1712	0.1785	0.6114
32	α-longipinene	C_15_H_24_	-	-	0.0065	0.0117	0.033
33	trans-Isoeugenol	C_10_H_12_O_2_	-	-	0.0186	0.0242	0.0411
34	(6,6-Dimethylbicyclo[3.1.1]hept-2-en-2-yl)methyl ethyl carbonate	C_13_H_20_O_3_	-	-	-	0.0077	-
35	Geranyl isovalerate	C_15_H_26_O_2_	-	-	0.0104	0.0201	0.0275
36	(−)-Spathulenol	C_15_H_24_O	0.0095	0.0053	0.0269	0.086	0.1504
37	Isospathulenol	C_15_H_24_O	-	-	0.0056	0.0209	0.0305
38	11,14-Eicosadienoic acid-methyl ester	C_21_H_38_O_2_	-	-	-	-	0.0035
39	9-Hexadecenoic acid	C_16_H_30_O_2_	-	-	-	0.0012	0.0169
40	Diethyl Phthalate	C_12_H_14_O_4_	0.0608	0.0054	0.0961	0.1208	0.0422
41	(6-Hydroxymethyl-2,3-dimethylphenyl)methanol	C_10_H_14_O_2_	0.024	0.0375	0.1359	0.3035	0.4167
42	Megastigma-4,6(E),8(Z)-triene	C_13_H_20_	-	-	-	-	0.0475
43	(Z)-3-butylidenephthalide	C_12_H_12_O_2_	0.0741	0.0659	0.3629	0.9156	1.3462
44	3-Butylisobenzofuran-1(3H)-one	C_12_H_14_O_2_	0.0154	0.169	0.5855	0.7921	0.4364
45	(Z)-Ligustilide	C_12_H_14_O_2_	1.1631	1.3302	24.204	25.0157	72.2466
46	A Senkyunolide A	C_12_H_16_O_2_	0.0092	0.0308	1.2552	3.6762	1.6264
47	β-ylangene	C_15_H_24_	-	-	-	-	0.1312
48	(E)-Ligustilide	C_12_H_14_O_2_	0.2955	0.6447	6.7875	21.9343	10.5147
49	9-Octadecenoic acid (Z)-, methyl ester	C_19_H_36_O_2_	-	-	0.0124	0.003	-
50	7,10-Octadecadienoic acid, methyl ester	C_19_H_34_O_2_	-	-	0.0745	0.124	-
51	Arteannuin b	C_15_H_20_O_3_	0.0388	0.0284	0.0506	0.2904	0.3535
52	Falcarinol	C_17_H_24_O	-	-	0.0082	0.0194	0.0818
53	Cedran-diol, 8S,13-	C_15_H_26_O_2_	-	-	0.0198	0.1405	0.1032
54	(Z,Z)-9,12-Octadecadienoic acid ethyl ester	C_20_H_36_O_2_	-	-	0.0751	0.0699	0.048
55	9,12-Octadecadienoic acid (Z,Z)-	C_18_H_32_O_2_	-	-	-	0.7065	-
56	1-Heptatriacotanol	C_37_H_76_O	-	-	0.0373	0.0744	0.0752
57	Panaxynone	C_17_H_22_O	-	0.013	0.3514	0.9662	0.8509
58	17-Pentatriacontene	C_35_H_70_	-	-	-	-	0.1297
59	γ-Sitosterol	C_29_H_50_O	-	-	-	0.0204	0.6237
60	2,2′-Methylenebis(6-tert-butyl-4-methylphenol)	C_23_H_32_O_2_	-	-	0.0201	0.0212	0.0379
61	Senkyunolide H	C_12_H_16_O_4_	-	-	-	0.9461	0.7792
62	Ethyl iso-allocholate	C_26_H_44_O_5_	-	0.032	0.0234	0.2136	0.2799
63	Vitamin E	C_29_H_50_O_2_	-	-	-	-	0.2918
64	Oligandrol	C_22_H_32_O_2_	-	-	-	0.176	0.2475
65	Stigmasterol	C_29_H_48_O	-	-	0.4304	0.541	1.1259

**Table 2:** Analysis of chemical components in the body of *A. sinensis* in different periods.

Number	Compounds	Formula	Relative Content (%)				
			April	June	August	September	October
1	9-Hexadecenoic acid	C_16_H_30_O_2_	-	-	-	0.0023	0.0018
2	9-Octadecen-12-ynoic acid methyl ester	C_19_H_32_O_2_	-	-	-	-	0.0019
3	3-Carene	C_10_H_16_	-	0.0041	0.005	0.0063	0.0605
4	α-Pinene	C_10_H_16_	0.002	0.0025	0.0211	0.05	0.2336
5	cis-β-Ocimene	C_10_H_16_	-	-	-	0.002	0.0047
6	1,2,6,6-tetramethylcyclohexa-1,3-diene	C_10_H_16_	-	0.0016	0.0012	0.0053	0.0109
7	5-Pentylcyclohexa-1,3-diene	C_11_H_18_	0.0034	0.088	0.0575	0.0524	0.2489
8	Pentylbenzene	C_11_H_16_	-	-	-	-	0.0128
9	p-Cymene	C_10_H_14_	-	0.0016	0.0016	0.0031	0.0036
10	Benzeneacetaldehyde, α-ethyl-	C_11_H_16_	-	-	-	0.0034	0.0104
11	Azulene	C_10_H_8_	-	0.0006	0.0017	0.0045	0.0038
12	3-methyl-5-propylnonane	C_13_H_28_	-	-	0.0019	0.0066	0.0256
13	Carveol	C_10_H_16_O	-	-	-	0.0012	0.0029
14	Naphthalene, 1,2,3,4-tetrahydro-1-methyl-	C_11_H_14_	-	-	0.0011	0.0062	0.0027
15	Limonen-6-ol, pivalate	C_15_H_24_O_2_	-	-	-	-	0.0024
16	6-Undecanone	C_11_H_22_O	-	0.0008	0.0046	0.0045	0.0273
17	Elemene isomer	C_15_H_24_	-	-	-	0.0074	0.037
18	2,5-Octadecadiynoic acid, methyl ester	C_19_H_30_O_2_	-	-	0.0006	0.003	0.0112
19	Tetradecane	C_14_H_30_	-	0.0077	0.0047	0.0084	0.0436
20	Bicyclo[4.4.1]undeca-1,3,5,7,9-pentaene	C_11_H_10_	-	-	0.0014	0.003	0.0027
21	α-acorenol	C_15_H_26_O	-	0.0017	0.0017	0.0021	0.01
22	(3R,3aR,7R,8aS)-3,8,8-Trimethyl-6-methyleneoctahydro-1H-3a,7-methanoazulene	C_15_H_24_	-	-	0.0128	0.0249	0.0786
23	β-copaene	C_15_H_24_	-	-	0.0027	0.0038	0.0086
24	Thujopsene-(I2)	C_15_H_24_	-	-	-	0.0009	0.0049
25	2-Methoxy-4-vinylphenol	C_9_H_10_O_2_	0.0068	0.0084	0.1304	0.0829	0.0895
26	cis-β-Farnesene	C_15_H_24_	-	-	-	0.0291	0.053
27	(±)-Gymnomitrene	C_15_H_24_	-	-	-	-	0.0917
28	α-Himachalene	C_15_H_24_	-	0.0109	0.03	0.0264	0.0726
29	β-Chamigrene	C_15_H_24_	-	-	0.0095	0.0101	0.0358
30	Patchoulene	C_15_H_24_	-	-	0.0036	0.0057	0.0189
31	β-Bisabolene	C_15_H_24_	-	-	0.0104	0.0245	0.0695
32	(+)-Bicyclogermacrene	C_15_H_24_	-	-	0.1502	0.1078	0.7927
33	(+)-Cuparene	C_15_H_22_	-	-	-	0.016	0.0125
34	α-longipinene	C_15_H_24_	-	-	0.006	0.012	0.0469
35	trans-Isoeugenol	C_10_H_12_O_2_	-	0.0146	0.0146	0.0176	0.0214
36	(6,6-Dimethylbicyclo[3.1.1]hept-2-en-2-yl)methyl ethyl carbonate	C_13_H_20_O_3_	-	-	-	0.0014	0.0135
37	Geranyl isovalerate	C_15_H_26_O_2_	-	-	0.0036	0.0057	0.0193
38	(−)-Spathulenol	C_15_H_24_O	0.0072	0.0029	0.0282	0.0323	0.1886
39	Isospathulenol	C_15_H_24_O	-	-	0.0052	0.0066	0.0431
40	11,14-Eicosadienoic acid-methyl ester	C_21_H_38_O_2_	-	-	-	0.0027	0.0066
41	9-Hexadecenoic acid	C_16_H_30_O_2_	-	-	-	-	0.0087
42	Diethyl Phthalate	C_12_H_14_O_4_	0.0089	0.006	0.0676	0.0771	0.03
43	(6-Hydroxymethyl-2,3-dimethylphenyl)methanol	C_10_H_14_O_2_	0.0126	0.0112	0.0348	0.0297	0.248
44	Megastigma-4,6(E),8(Z)-triene	C_13_H_20_	-	-	-	-	0.0678
45	(Z)-3-butylidenephthalide	C_12_H_12_O_2_	0.0667	0.0432	0.2299	0.35	0.9806
46	3-Butylisobenzofuran-1(3H)-one	C_12_H_14_O_2_	0.0139	0.0231	0.1442	0.2048	0.5095
47	(Z)-Ligustilide	C_12_H_14_O_2_	1.187	0.9286	15.5991	16.3184	71.3681
48	Senkyunolide A	C_12_H_16_O_2_	0.062	0.0427	0.4202	0.3677	1.5863
49	β-ylangene	C_15_H_24_	-	-	-	-	0.0287
50	(E)-Ligustilide	C_12_H_14_O_2_	0.3673	0.4558	3.7996	8.357	9.0088
51	9-Octadecenoic acid (Z)-, methyl ester	C_19_H_36_O_2_	-	-	0.0049	0.0121	0.0921
52	7,10-Octadecadienoic acid, methyl ester	C_19_H_34_O_2_	-	-	0.0145	0.0292	0.2559
53	Arteannuin b	C_15_H_20_O_3_	0.0525	0.0167	0.0704	0.1144	0.2202
54	cis-5,8,11,14,17-Eicosapentaenoic acid	C_20_H_30_O_2_	-	-	-	-	0.0032
55	Falcarinol	C_17_H_24_O	-	-	0.0141	0.0229	0.0851
56	Cedran-diol, 8S,13-	C_15_H_26_O_2_	-	-	0.0093	0.0343	0.0504
57	(Z,Z)-9,12-Octadecadienoic acid ethyl ester	C_20_H_36_O_2_	-	-	0.0255	0.0365	0.1807
58	9,12-Octadecadienoic acid (Z,Z)-	C_18_H_32_O_2_	-	-	0.0043	0.246	3.607
59	1-Heptatriacotanol	C_37_H_76_O	-	-	0.0167	0.0574	0.077
60	Panaxynone	C_17_H_22_O	-	0.017	0.1677	0.668	2.634
61	γ-Sitosterol	C_29_H_50_O	-	-	0.4142	0.3163	1.8592
62	2,2′-Methylenebis(6-tert-butyl-4-methylphenol)	C_23_H_32_O_2_	-	0.012	0.012	0.0283	0.0065
63	Senkyunolide H	C_12_H_16_O_4_				0.0386	0.1186
64	Ethyl iso-allocholate	C_26_H_44_O_5_		0.0050	0.0050	0.0592	0.0629
65	E Vitamin E	C_29_H_50_O_2_			0.0951	0.1653	0.2143
66	Oligandrol	C_22_H_32_O_2_			0.0473	0.1613	0.1873
67	Campesterol	C_28_H_48_O					0.0463
68	Stigmasterol	C_29_H_48_O			0.2867	0.0651	0.8698

**Table 3:** Analysis of chemical composition in the tail of *A. sinensis* in different periods.

Number	Compounds	Formula	Relative Content (%)				
			April	June	August	September	October
1	9-Hexadecenoic acid	C_16_H_30_O_2_	-	-	0.001	0.001	0.0079
2	9-Octadecen-12-ynoic acid methyl ester	C_19_H_32_O_2_	-	-	-	-	0.0015
3	3-Carene	C_10_H_16_	-	0.0019	0.0033	0.0056	0.0134
4	α-Pinene	C_10_H_16_	0.0008	0.0082	0.0849	0.1553	0.523
5	cis-β-Ocimene	C_10_H_16_	-	-	0.0013	0.0032	0.01
6	10,13-Octadecadiynoic acid	C_19_H_30_O_2_	-	-	-	-	0.0007
7	1,2,6,6-tetramethylcyclohexa-1,3-diene	C_10_H_16_	-	0.0022	0.0062	0.011	0.0226
8	5-Pentylcyclohexa-1,3-diene	C_11_H_18_	0.0012	0.0035	0.0714	0.0853	0.2548
9	Pentylbenzene	C_11_H_16_	-	-	-	-	0.0104
10	p-Cymene	C_10_H_14_	-	0.0016	0.0044	0.0036	0.0048
11	Benzeneacetaldehyde, α-ethyl-	C_11_H_16_	-	-	0.0043	0.0058	0.0125
12	Azulene	C_10_H_8_	-	0.0013	0.0035	0.0046	-
13	3-methyl-5-propylnonane	C_13_H_28_	-	-	0.0036	0.0063	-
14	Carveol	C_10_H_16_O	-	-	0.0054	0.0053	0.0149
15	Naphthalene, 1,2,3,4-tetrahydro-1-methyl-	C_11_H_14_	-	-	-	0.0054	-
16	Limonen-6-ol, pivalate	C_15_H_24_O_2_	-	-	0.0037	0.0034	0.0101
17	6-Undecanone	C_11_H_22_O	-	0.0029	0.0053	0.0059	0.0194
18	Elemene isomer	C_15_H_24_	-	-	-	0.0088	0.0358
19	2,5-Octadecadiynoic acid, methyl ester	C_19_H_30_O_2_	-	-	0.002	0.003	0.0079
20	Tetradecane	C_14_H_30_	-	0.0038	0.0063	0.0064	0.0154
21	α-Elemene	C_15_H_24_	-	-	-	-	0.0059
22	Bicyclo[4.4.1]undeca-1,3,5,7,9-pentaene	C_11_H_10_	-	0.0009	0.0014	0.003	0.005
23	Dicyclopentadiene diepoxide	C_10_H_12_O_2_	-	-	-	0.0011	0.0045
24	α-acorenol	C_15_H_26_O	-	0.0018	0.0055	0.0028	0.0132
25	(3R,3aR,7R,8aS)-3,8,8-Trimethyl-6-methyleneoctahydro-1H-3a,7-methanoazulene	C_15_H_24_	-	-	0.0613	0.0547	0.1687
26	β-copaene	C_15_H_24_	-	0.0089	0.0077	0.0085	0.0234
27	Thujopsene-(I2)	C_15_H_24_	-	-	-	0.0038	0.016
28	2-Methoxy-4-vinylphenol	C_9_H_10_O_2_	0.0045	0.0376	0.0833	0.0814	0.0862
29	cis-β-Farnesene	C_15_H_24_	-	-	0.042	0.0478	0.1137
30	(±)-Gymnomitrene	C_15_H_24_	-	-	0.0548	0.0778	0.1954
31	α-Himachalene	C_15_H_24_	-	0.0088	0.0402	0.0443	0.1269
32	β-Chamigrene	C_15_H_24_	-	0.0039	0.0322	0.0244	0.0924
33	gleenol	C_14_H_20_O	-	-	-	-	0.0021
34	Patchoulene	C_15_H_24_	-	-	0.0204	0.0159	0.0479
35	β-Guaiene	C_15_H_24_	-	-	-	-	0.0009
36	β-Bisabolene	C_15_H_24_	-	-	0.0668	0.0549	0.2088
37	(+)-Bicyclogermacrene	C_15_H_24_	-	-	0.1905	0.1685	0.9263
38	(+)-Cuparene	C_15_H_22_	-	-	-	0.0244	0.0406
39	α-longipinene	C_15_H_24_	-	-	0.0386	0.0464	0.1153
40	trans-Isoeugenol	C_10_H_12_O_2_	-	0.003	0.0126	0.0066	0.0205
41	(6,6-Dimethylbicyclo[3.1.1]hept-2-en-2-yl)methyl ethyl carbonate	C_13_H_20_O_3_	-	-	-	-	0.0034
42	Geranyl isovalerate	C_15_H_26_O_2_	-	-	0.0025	0.0065	0.0114
43	(−)-Spathulenol	C_15_H_24_O	0.0015	0.0087	0.0686	0.0534	0.2948
44	Isospathulenol	C_15_H_24_O	-	-	0.0164	0.0114	0.0524
45	11,14-Eicosadienoic acid-methyl ester	C_21_H_38_O_2_	-	-	0.0031	0.0017	0.003
46	9-Hexadecenoic acid	C_16_H_30_O_2_	-	-	-	-	0.0076
47	Diethyl Phthalate	C_12_H_14_O_4_	0.0027	0.0284	0.0274	0.2252	0.0168
48	(6-Hydroxymethyl-2,3-dimethylphenyl)methanol	C_10_H_14_O_2_	-	0.0415	0.0141	0.0347	0.0914
49	Megastigma-4,6(E),8(Z)-triene	C_13_H_20_	-	-	-	0.0307	0.0666
50	(Z)-3-butylidenephthalide	C_12_H_12_O_2_	0.0184	0.0908	0.3368	0.536	0.8135
51	3-Butylisobenzofuran-1(3H)-one	C_12_H_14_O_2_	0.0058	0.038	0.2739	0.2837	0.3314
52	(Z)-Ligustilide	C_12_H_14_O_2_	0.3308	3.0916	27.5304	42.1721	73.4925
53	Senkyunolide A	C_12_H_16_O_2_	0.024	0.0783	0.7661	1.0886	1.4591
54	β-ylangene	C_15_H_24_	-	-	-	-	0.0477
55	(E)-Ligustilide	C_12_H_14_O_2_	0.0823	1.5362	9.2198	17.4163	9.6689
56	9-Octadecenoic acid (Z)-, methyl ester	C_19_H_36_O_2_	-	0.0008	0.0069	0.002	0.0275
57	7,10-Octadecadienoic acid, methyl ester	C_19_H_34_O_2_	-	0.0264	0.02	0.0576	0.0436
58	Arteannuin b	C_15_H_20_O_3_	0.0088	0.0208	0.0549	0.1587	0.1896
59	Falcarinol	C_17_H_24_O	-	-	0.0409	0.1145	0.1906
60	Cedran-diol, 8S,13-	C_15_H_26_O_2_	-	-	0.0194	0.0454	0.0734
61	(Z,Z)-9,12-Octadecadienoic acid ethyl ester	C_20_H_36_O_2_	-	-	0.0178	0.0717	0.0641
62	9,12-Octadecadienoic acid (Z,Z)-	C_18_H_32_O_2_	-	-	0.0106	0.6222	2.5576
63	1-Heptatriacotanol	C_37_H_76_O	-	0.004	0.044	0.0839	0.1142
64	Panaxynone	C_17_H_22_O	-	0.1361	0.9783	4.6962	2.5576
65	17-Pentatriacontene	C_35_H_70_	-	0.0277	-	-	0.1284
66	γ-Sitosterol	C_29_H_50_O	-	-	0.2192	1.9209	0.5882
67	2,2′-Methylenebis(6-tert-butyl-4-methylphenol)	C_23_H_32_O_2_	-	0.0078	0.0243	0.0267	0.0481
68	Senkyunolide H	C_12_H_16_O_4_	-	-	-	0.0397	0.0413
69	Ethyl iso-allocholate	C_26_H_44_O_5_	-	0.02	0.0646	0.1017	0.0015
70	Vitamin E	C_29_H_50_O_2_	-	-	0.0087	0.1275	0.1838
71	Oligandrol	C_22_H_32_O_2_	-	-	0.1583	0.322	0.1814
72	Stigmasterol	C_29_H_48_O	-	-	0.1827	0.4067	0.4229

**Table 4:** The number of chemical compounds of *A. sinensis* in different parts in different periods.

Medicinal Parts of *A. sinensis*	April	June	August	September	October
Head	12	20	42	53	61
Body	12	24	48	59	68
Tail	11	31	55	62	69

## Data Availability

The data presented in this study are available on request from the corresponding author.

## Sample Availability

Samples of the plant materials of *Angelica sinensis* are available from the authors.
